# Comparison of effectiveness of four myopia control interventions in Chinese children: a real-world retrospective study

**DOI:** 10.1186/s40662-026-00487-z

**Published:** 2026-05-12

**Authors:** Lizhen Chen, Li Sun, Xiaojing Chen, Jiaxing Liu, Jinfa Li, Hiu Ying Leung, Jingfa Zhang, Dennis S. C. Lam

**Affiliations:** 1 Department of Ophthalmology, Zhujiang Hospital, Southern Medical University, Guangzhou, China; 2 The Primasia International Eye Research Institute (PIERI), School of Medicine, The Chinese University of Hong Kong (Shenzhen), Shenzhen, China; 3 Department of Ophthalmology and Visual Sciences, The Chinese University of Hong Kong, Hong Kong, China; 4 Eye Department, C+ Health CKJ (Shenzhen) Hospital, Shenzhen, China; 5 C-MER (Guangzhou) Dennis Lam Eye Hospital, Guangzhou, China; 6 C-MER (Shenzhen) Dennis Lam Eye Hospital, Shenzhen, China; 7 The C-MER Dennis Lam and Partners Eye Center, C-MER International Eye Care Group, Hong Kong, China

**Keywords:** Myopia control, Axial length, Defocus incorporated multiple segments, Highly aspherical lenslets, Low-concentration atropine, Combination therapy, Real-world study

## Abstract

**Background:**

Myopia is a global public health issue, necessitating effective control strategies. Defocus incorporated multiple segments (DIMS) and highly aspherical lenslet (HAL) spectacle lenses, as well as their combination with low-concentration atropine (0.01%), have been shown to control myopia effectively. However, direct comparisons among these regimens, DIMS, HAL, DIMSA (DIMS + atropine), and HALA (HAL + atropine), are lacking. In this real-world study, we compared their effectiveness in slowing myopia progression.

**Methods:**

This single-center retrospective cohort study included 347 children (694 eyes) aged 6–14 years who received one of the four interventions for at least 1 year. The primary outcome was the annual change in axial length (AL). The secondary outcome was the annual change in spherical equivalent refraction (SER). The treatment response was categorized as good, fair, or poor. Linear mixed models and generalized estimating equations were used for analysis, adjusting for covariates.

**Results:**

After adjustment, annual axial elongation differed significantly among the groups (*P* < 0.001), with marginal means of 0.23 mm (DIMS), 0.15 mm (HAL), 0.20 mm (DIMSA), and 0.12 mm (HALA). Post hoc comparisons revealed that both HAL and HALA were superior to DIMS (*P* < 0.001) and that HALA was superior to DIMSA (*P* = 0.022). Adding 0.01% atropine to either optical monotherapy did not provide a significant additional benefit in terms of mean AL or SER outcomes. Compared with DIMS, HALA significantly increased the odds of a good response for AL (OR = 4.34, *P* < 0.001) and SER (OR = 5.85, *P* < 0.001), whereas this effect was not observed with DIMSA. Older age independently predicted better response (AL: OR = 1.46/year; SER: OR = 1.26/year; both *P* < 0.001).

**Conclusions:**

In this non-randomized real-world comparison, HAL monotherapy was associated with slower SER progression, compared with DIMS. Although adding atropine did not improve mean outcomes, HALA showed the highest likelihood of a good response, suggesting a clinically meaningful benefit in response distribution. Although HALA did not consistently outperform HAL monotherapy across analyses, its advantage in achieving favourable individual responses supports its consideration for children requiring maximal intervention. These findings require confirmation in prospective randomized trials.

**Supplementary Information:**

The online version contains supplementary material available at 10.1186/s40662-026-00487-z.

## Background

Myopia has become a global public health concern, highlighting the urgent need for effective interventions to slow its progression and prevent vision-threatening complications in children [1]. Among the available strategies, optical interventions based on defocus modification technology have shown significant efficacy [2]. Spectacle lenses, such as defocus incorporated multiple segments (DIMS) and highly aspherical lenslets (HAL), are leading non-invasive options; both have demonstrated significant efficacy in slowing axial elongation and refractive progression, compared with single-vision lenses [3].

However, direct comparisons between DIMS and HAL have yielded variable results, potentially owing to differences in study population, study design, and follow-up duration. A 2-year study in a European cohort reported that DIMS and HAL were essentially equivalent in controlling myopia [4]. However, a larger real-world study from the same population found that HAL was statistically superior to DIMS, although the difference was not clinically significant [5]. In contrast, other studies, primarily involving Chinese populations, have suggested that HAL may provide better control of axial elongation than DIMS does [6]. Consistent with this finding, our previous study showed that HAL monotherapy was more effective than DIMS monotherapy, particularly in younger children [7].

To achieve greater treatment effects, attention has shifted towards combination strategies. One such approach involves supplementing optical interventions with low-concentration atropine (0.01%). Compared with monotherapy, the combination of atropine with DIMS has shown enhanced effectiveness [8–12]. Similarly, preliminary evidence has emerged, showing that the combination of atropine with HAL provides additional benefit over HAL monotherapy [13, 14]. However, it remains unclear whether the established superiority of HAL over DIMS as a monotherapy persists in the context of combination therapy. The current literature lacks a direct comparison of these four strategies [9, 15]. Such a comparison is essential for guiding personalised treatment decisions, particularly given the potential for differential synergistic effects between the distinct optical designs of DIMS and HAL and atropine.

Therefore, to address this gap in the literature and guide personalised clinical decision-making, in this study, we aimed to directly compare all four key regimens, DIMS, HAL, DIMS with 0.01% atropine (DIMSA), and HAL with 0.01% atropine (HALA), within a single large real-world cohort. By evaluating whether the monotherapy advantage of HAL over DIMS is maintained in combination with atropine and identifying the most effective overall strategy during the first year of treatment, this study sought to provide preliminary evidence to inform the optimisation of myopia management in children.

## Methods

### Study design

This was a single-center, retrospective, real-world cohort study conducted at C-MER (Shenzhen) Dennis Lam Eye Hospital. Data were extracted from the electronic medical record system from March 2021 to June 2025. The patient cohort in this study was entirely independent and non-overlapping with that of our previous publication [7]. The study adhered to the tenets of the Declaration of Helsinki and was approved by the Ethics Review Committee of the Second School of Clinical Medicine, Southern Medical University (Approval No: 2025-KY-171). The requirement for informed consent was waived given the retrospective nature of the study.

### Participants

The study participants were children and adolescents aged 6–14 years who continuously received one of the four interventions, DIMS, HAL, DIMSA, and HALA, for at least 1 year. The participants were divided into four groups based on the treatment strategies, namely the DIMS, HAL, DIMSA and HALA groups. The DIMSA and HALA groups received 0.01% atropine eye drops as combination therapy.

The inclusion criteria for the study were the following: 1) age of 6–14 years; 2) best-corrected visual acuity (BCVA) ≥ 0.1 logMAR; 3) good compliance with one of the four interventions being reported (compliance with spectacles was defined as reported full-time wear during waking hours; compliance with atropine was defined as parent-reported administration of one drop per eye at bedtime, ≥ 5 days per week on average); 4) complete baseline and follow-up data being obtained for axial length (AL), spherical equivalent refraction (SER), cylinder power, corneal astigmatism, and corneal curvature, with a follow-up interval of at least 11 months.

The exclusion criteria included (1) use of other myopia control interventions, including orthokeratology, multifocal soft contact lenses, or repeated low-level red light therapy, for > 3 months before enrolment or during the follow-up period and (2) presence of any ocular disease, including strabismus, amblyopia, congenital cataracts, glaucoma, retinopathy, or other ocular or systemic abnormalities.

### Data collection

As part of routine clinical practice, all patients underwent comprehensive ophthalmic examinations at the baseline and follow-up visits. These examinations followed standardized clinical protocols, which are described below. For this retrospective study, the results of these examinations were subsequently retrieved from the electronic medical record system, and no study-specific examinations or procedures were performed.

BCVA was assessed using the International Standard Logarithmic Visual Acuity Chart. AL was measured with an optical biometer (IOLMaster 700, Carl Zeiss, Germany), and the average of three repeated measurements for each eye was used for analysis. Corneal curvature and astigmatism were measured using an automated refractometer (TonoRef II, Nidek, Japan). For non-cycloplegic refraction, the fogging technique was employed to minimize residual accommodation: the eye was fogged with + 1.00 diopter (D) spherical over-correction until vision blurred, then slowly reduced until the patient achieved BCVA. The endpoint was determined as the maximum plus sphere that provided the best visual acuity, that is, the highest corrected distance visual acuity with the least minus power. Cycloplegic refraction was performed using 1% cyclopentolate, administered twice per eye at 5-min intervals, with refraction measured 30 min after the last instillation. Cylinder power was recorded as a positive value. Slit-lamp examination and fundus evaluation were performed to rule out pathological changes.

### Outcomes

#### Primary outcome

The annual AL change (mm/year), defined as the change in AL from baseline to follow-up, was standardized to a 365-day period. The annual AL change was calculated as follows: annual AL change = (AL [follow-up] – AL [baseline]) / interval (in days) × 365.

#### Secondary outcomes

SER was calculated as the spherical power plus half of the negative cylinder power. The annual SER change (D/year) was derived as follows: (SER [follow-up] – SER [baseline])/interval (in days) × 365.

For the calculation of annual SER change, a consistent measurement method was applied within each individual: if cycloplegic refraction was performed at the baseline and follow-up visits, the cycloplegic measurements were used for both time points. For all other individuals, non-cycloplegic refraction, performed using the fogging technique, was used for the baseline and follow-up visits to ensure internal consistency in the calculation of annual SER change.

Treatment response was categorized as good, fair, or poor based on predefined thresholds for annual changes in AL and SER, selected based on clinical consensus and previously published criteria.

- For AL, the ≤ 0.20 mm/year cutoff (good response) corresponds to the physiological axial elongation rate in age-matched emmetropic children [16–18], whereas the ≥ 0.40 mm/year cutoff (poor response) represents clinically significant progression associated with an increased risk of high myopia [19].

Good response: ≤ 0.20 mm/year

Fair response: 0.20 to 0.40 mm/year

Poor response: ≥ 0.40 mm/year

- For SER, the ≥ − 0.50 D/year cutoff (good response) aligns with the minimal clinically important difference in myopia progression [16, 20], and the ≤ − 0.75 D/year cutoff (poor response) represents rapid progression warranting treatment escalation [19, 21].

Good response: ≥ − 0.5 D/year

Fair response: − 0.5 to − 0.75 D/year

Poor response: ≤ − 0.75 D/year

### Statistical analysis

All statistical analyses were performed using SPSS 30 (IBM Corp., Armonk, NY, USA). A two-sided *P* value < 0.05 was considered statistically significant for all analyses.

### Baseline characteristics

Participant-level analysis was performed using data from the right eye only. Continuous variables were presented as means ± standard deviations (SDs) or medians (interquartile ranges), depending on the normality of their distribution, assessed using the Shapiro–Wilk test. Group comparisons were made using one-way analysis of variance or the Kruskal–Wallis test, as appropriate. Categorical variables were presented as counts (percentages) and compared using the chi-square test.

Given the hierarchical structure of our data, with two eyes nested within each participant, observations from the same individual are correlated and cannot be treated as independent. To account for this correlation while utilizing all available data, we employed linear mixed models (LMM) for continuous outcomes and generalized estimating equations (GEE) for binary outcomes.

### Primary analysis

To utilize all available data and account for within-participant interocular correlation, the primary analysis included data from both eyes of all 347 participants (694 eyes) using LMM. A random intercept for each participant was incorporated. The fixed effects included the treatment group and covariates of sex, age, follow-up interval, and baseline ocular parameters (AL, SER, cylinder power, corneal astigmatism, and corneal curvature). Post hoc pairwise comparisons were performed with Bonferroni correction. The results are presented as adjusted marginal means ± standard errors (SEs).

### Treatment response analysis

To enhance statistical power and focus on the most clinically relevant contrast, all included eyes were classified as those having either a good response or non-good response (combining fair and poor responses). GEE was used to compare the likelihood of a good response across the treatment groups after adjustment for the same covariates as those in the primary analysis. The odds ratios (ORs) with 95% confidence intervals (CIs) are reported.

### Sensitivity analysis

To ensure that the findings were not unduly dependent on these more complex modelling choices and to confirm the primary findings, a sensitivity analysis was performed using analysis of covariance (ANCOVA) on the data from the right eyes only (n = 347), adjusting for the same covariates. The right eyes were selected a priori to avoid potential bias arising from interocular correlation and to maintain consistency with the baseline comparisons, which were also performed using the right-eye data.

## Results

### Baseline characteristics

A total of 347 children (694 eyes) were included in this study. Their mean ± SD age at baseline was 10.35 ± 2.02 years, and 53.0% (184/347) were male, and there were no significant differences in the sex distribution across the treatment groups (χ^2^ = 1.04, *P* = 0.792). Analysis of the right-eye data revealed that baseline AL (*P* = 0.139) and corneal curvature (F = 1.82, *P* = 0.143) were comparable across the groups, whereas significant intergroup differences were observed in age, follow-up interval, baseline SER, cylinder power, and corneal astigmatism (all *P* < 0.05). The initial mean ± SD AL and SER in the right eyes were 24.67 ± 1.06 mm (range, 21.93 to 28.43 mm) and − 2.55 ± 1.81 D (range, − 7.72 to 0.54 D), respectively. Detailed baseline demographics are summarized in Table 1.

**Table 1:** Comparison of baseline characteristics among the treatment groups

Characteristic		DIMS (n = 122)	HAL (n = 94)	DIMSA (n = 73)	HALA (n = 58)	*P*
Age (years)		10.79 (8.50, 12.43)	10.75 (8.81, 12.10)	9.98 (8.88, 11.07)	9.86 (8.23, 11.01)	0.031
Sex (M/F)		65/57	48/46	37/36	34/24	0.792
Follow-up interval (years)		1.47 (1.04, 2.00)	1.04 (0.92, 1.22)	1.07 (0.98, 1.28)	1.10 (0.98, 1.28)	< 0.001
Axial length (mm)	OD	24.60 (24.01, 25.55)	24.43 (23.76, 24.95)	24.55 (24.00, 25.40)	24.47 (23.79, 25.18)	0.139
	OS	24.64 (24.02, 25.37)	24.54 (23.89, 25.13)	24.54 (23.99, 25.24)	24.42 (23.79, 25.24)	-
Spherical equivalent refraction (D)	OD	− 2.46 (− 4.29, − 1.56)	− 1.64 (− 2.59, − 1.14)	− 1.96 (− 3.43, − 1.30)	− 1.75 (− 4.06, − 0.99)	< 0.001
	OS	− 2.22 (− 3.34, − 1.40)	− 1.89 (− 2.78, − 0.95)	− 1.94 (− 3.16, − 1.03)	− 1.83 (− 3.73, − 1.25)	-
Cylinder power (D)	OD	0.82 (0.40, 1.55)	0.53 (0.26, 1.05)	0.76 (0.37, 1.15)	0.55 (0.30, 1.17)	0.024
	OS	0.99 (0.44, 1.77)	0.67 (0.36, 1.12)	0.79 (0.51, 1.23)	0.76 (0.32, 1.57)	-
Corneal astigmatism (D)	OD	1.37 (0.93, 2.00)	1.04 (0.65, 1.63)	1.25 (0.94, 1.59)	1.00 (0.63, 1.68)	0.005
	OS	1.53 (1.09, 2.11)	1.19 (0.81, 1.69)	1.31 (0.92, 1.66)	1.26 (0.65, 1.77)	-
Corneal curvature (D)	OD	43.24 ± 1.43	43.17 ± 1.39	42.80 ± 1.48	43.30 ± 1.38	0.143
	OS	43.26 ± 1.41	43.14 ± 1.34	42.78 ± 1.51	43.30 ± 1.44	-

The baseline demographic and ocular parameters of both eyes are presented, whereas only the right-eye data were used for baseline comparisons

*DIMS *= defocus incorporated multiple segments; *DIMSA *= DIMS with 0.01% atropine; *F* = female; *HAL =* highly aspherical lenslets; *HALA *= HAL with 0.01% atropine; *M *= male; *OD *= right eye ; *OS * = left eye

### Primary outcome: annual axial elongation was lower in the HAL and HALA groups

**Fig. 1:**
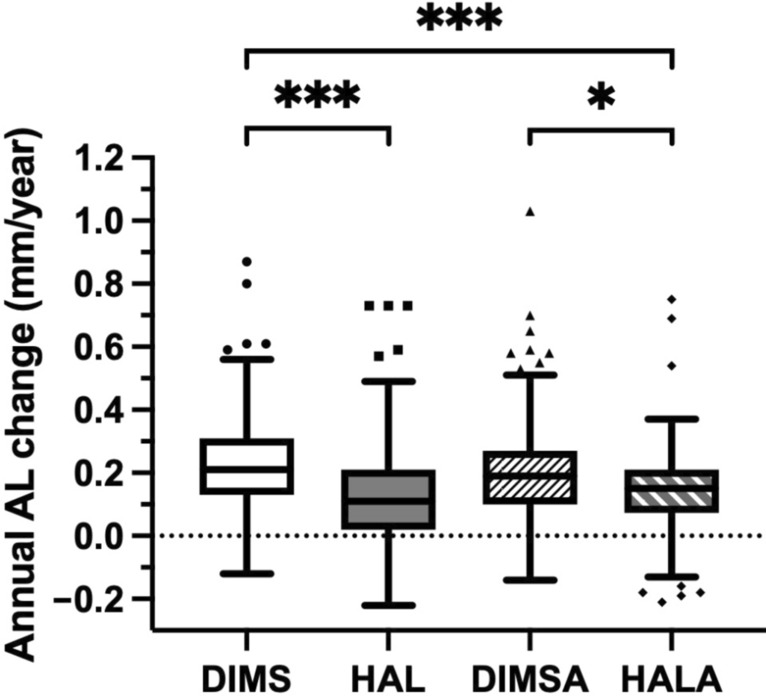
Group comparisons of annual axial elongation. The data are presented as Tukey-style box plots showing the median (center line), interquartile range (IQR, box), and 1.5 × IQR (whiskers). The linear mixed model, adjusted for age, sex, follow-up interval, and baseline ocular parameters, identified a significant treatment effect (*P* < 0.001). Post hoc pairwise comparisons with Bonferroni correction revealed that axial elongation was significantly slower in the HAL (n = 188**)** and HALA (n = 116) groups than in the DIMS (n = 244) group, and in the HALA group than in the DIMSA (n = 146) group. ***P < 0.001; *P = 0.022. AL, axial length; DIMS, defocus incorporated multiple segments; DIMSA, DIMS with 0.01% atropine; HAL, highly aspherical lenslets; HALA, HAL with 0.01% atropine

The unadjusted mean ± SD annual axial elongations were 0.23 ± 0.15 mm, 0.13 ± 0.17 mm, 0.20 ± 0.17 mm, and 0.14 ± 0.15 mm in the DIMS, HAL, DIMSA, and HALA groups, respectively. The LMM, adjusted for age, sex, follow-up interval, and baseline ocular parameters (AL, SER, cylinder power, corneal astigmatism, and corneal curvature), revealed a significant effect of treatment on axial elongation (F (3, 331) = 8.74, *P* < 0.001). The adjusted marginal means ± SE were 0.23 ± 0.01 mm, 0.15 ± 0.02 mm, 0.20 ± 0.02 mm, and 0.12 ± 0.02 mm for the DIMS, HAL, DIMSA, and HALA groups, respectively (Table 2 and Fig. 1).

**Table 2:** Group comparisons of unadjusted and adjusted annual change in AL and SER

Group	AL (mm)		SER (D)	
	Unadjusted*	Adjusted**	Unadjusted*	Adjusted**
DIMS	0.23 ± 0.15	0.23 ± 0.01	− 0.42 ± 0.33	− 0.42 ± 0.03
HAL	0.13 ± 0.17	0.15 ± 0.02	− 0.22 ± 0.39	− 0.25 ± 0.04
DIMSA	0.20 ± 0.17	0.20 ± 0.02	− 0.32 ± 0.36	− 0.32 ± 0.04
HALA	0.14 ± 0.15	0.12 ± 0.02	− 0.17 ± 0.34	− 0.15 ± 0.04

*AL *= axial length; *DIMS *= defocus incorporated multiple segments; *DIMSA *= DIMS with 0.01% atropine; *HAL *= highly aspherical lenslets; *HALA *= HAL with 0.01% atropine; *LMM *= linear mixed model; SER = spherical equivalent refraction

^*^ Unadjusted data are presented as mean ± standard deviation; **Adjusted data of marginal mean ± standard error are derived from the LMM

Post hoc pairwise comparisons with Bonferroni correction revealed that the HAL and HALA groups demonstrated significantly slower axial elongation than the DIMS group did (mean difference [MD]: 0.08 mm, 95% CI: 0.03 to 0.14 mm, *P* < 0.001 and 0.11 mm, 95% CI: 0.04 to 0.17 mm, *P* < 0.001, respectively). Furthermore, HALA was significantly more effective than DIMSA (MD: 0.07 mm, 95% CI: 0.01 to 0.14 mm, *P* = 0.022; Table 3). However, adding 0.01% atropine to either DIMS or HAL did not provide a significant additional benefit (DIMS vs. DIMSA: *P* = 0.758; HAL vs. HALA: *P* = 1.000).

**Table 3:** Significant results from post hoc pairwise comparisons of annual change in AL and SER

Comparison	AL (mm)			SER (D)		
	MD	95% CI	*P*	MD	95% CI	*P*
DIMS vs. HAL	0.08	0.03 to 0.14	< 0.001	− 0.17	− 0.30 to − 0.04	0.004
DIMS vs. HALA	0.11	0.04 to 0.17	< 0.001	− 0.27	− 0.41 to − 0.12	< 0.001
DIMSA vs. HALA	0.07	0.01 to 0.14	0.022	− 0.17	− 0.32 to − 0.02	0.020

*AL *= axial length; *CI *= confidence interval; *DIMS *= defocus incorporated multiple segments; DIMSA = DIMS with 0.01% atropine; *HAL *= highly aspherical lenslets; *HALA *= HAL with 0.01% atropine; *MD *= mean difference; *SER *= spherical equivalent refraction

Post hoc pairwise comparisons with Bonferroni correction. Only comparisons with significant results are shown

The LMM also identified older age (β = − 0.04 mm/year per year, *P* < 0.001) and a less negative baseline SER (lower myopia; β = − 0.02 mm/year per diopter, *P* = 0.011) as independent factors associated with slower axial elongation.

### HAL and HALA groups showed less SER progression

**Fig. 2:**
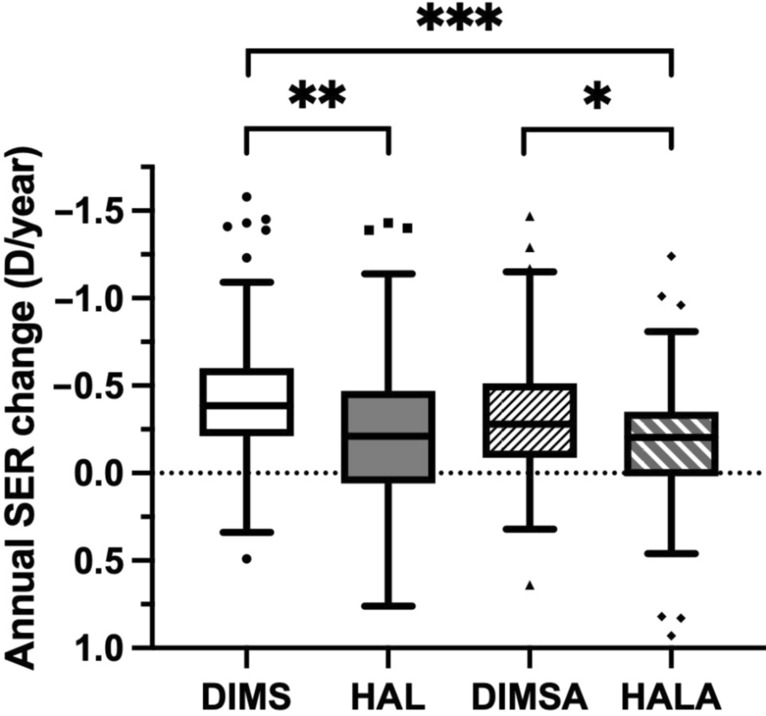
Group comparisons of annual SER changes. The data are presented as Tukey-style box plots showing the median (center line), interquartile range (IQR, box), and 1.5 × IQR (whiskers). The linear mixed model, adjusted for the same covariates, identified a significant overall treatment effect (*P* < 0.001). Post hoc pairwise comparisons with Bonferroni correction revealed that SER progression was significantly lower in the HAL (n = 188) and HALA (n = 116) groups than in the DIMS (n = 244) group, and in the HALA group than in the DIMSA (n = 146) group. ***P < 0.001; **P = 0.004; *P = 0.020. DIMS, defocus incorporated multiple segments; DIMSA, DIMS with 0.01% atropine; HAL, highly aspherical lenslets; HALA, HAL with 0.01% atropine; SER, spherical equivalent refraction

The analysis of SER progression yielded a pattern of results fully consistent with the AL findings. The LMM identified a significant treatment effect on the annual change in SER (F (3, 331) = 8.94, *P* < 0.001). After adjustment, the marginal means ± SE were − 0.42 ± 0.03 D, − 0.25 ± 0.04 D, − 0.32 ± 0.04 D and − 0.15 ± 0.04 D in the DIMS, HAL, DIMSA and HALA groups, respectively. Post hoc pairwise comparisons with Bonferroni correction revealed that the HAL and HALA groups demonstrated significantly less SER progression than did the DIMS group (MD: − 0.17 D, 95% CI: − 0.30 to − 0.04 D, *P* = 0.004; MD: − 0.27 D, 95% CI: − 0.41 to − 0.12 D, *P* < 0.001). The HALA group also demonstrated superior outcomes, compared with the DIMSA group (MD: − 0.17 D, 95% CI: − 0.32 to − 0.02 D, *P* = 0.020; Table 3 and Fig. 2). No significant improvement was observed with atropine supplementation in either DIMS (*P* = 0.314) or HAL (*P* = 0.453). The LMM for SER progression also identified older age as a significant predictor of slower SER progression (β = 0.04 D/year, *P* < 0.001).

### HALA therapy was associated with the highest likelihood of a good treatment response

Treatment responses were evaluated using clinically significant thresholds for annual axial elongation (≤ 0.20 mm/year) and annual SER change (≥ − 0.50 D/year), with a good response defined according to these thresholds.

**Fig. 3:**
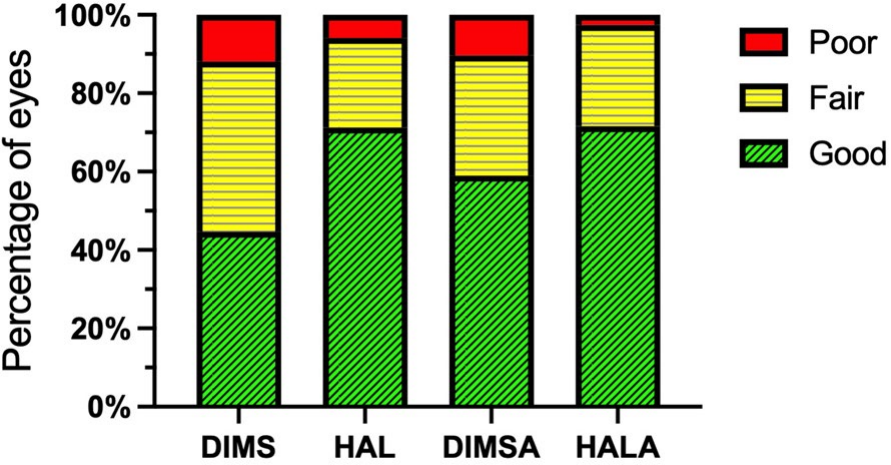
Distribution of treatment response based on axial elongation. Stacked bar charts illustrate the distribution of treatment response for each intervention group. Sample sizes (eyes): DIMS, n = 244; HAL, n = 188; DIMSA, n = 146; HALA, n = 116. Statistical analysis was performed using generalized estimating equation, which included data from all eligible eyes and adjusted for age, sex, follow-up interval, and baseline ocular parameters. The model identified a significant overall difference in the likelihood of achieving a good response among the groups (Wald χ^2^ = 24.10, *P* < 0.001). The HALA group demonstrated the highest rate of good response. DIMS, defocus incorporated multiple segments; DIMSA, DIMS with 0.01% atropine; HAL, highly aspherical lenslets; HALA, HAL with 0.01% atropine

The proportion of eyes with a good response based on axial elongation was highest in the HALA group (71.6%), followed by the HAL (71.3%), DIMSA (58.2%), and DIMS (44.7%) groups (Table 4 and Fig. 3). The GEE model confirmed a significant difference in the likelihood of a good response among the groups (Wald χ^2^ = 24.10, *P* < 0.001). Compared with DIMS, HALA was associated with the highest odds of a good response (OR = 4.34, 95% CI: 2.25 to 8.37, *P* < 0.001). The HAL and DIMSA interventions were also associated with significantly higher odds of achieving a good response than was DIMS (OR = 2.88, 95% CI: 1.61 to 5.14, *P* < 0.001 and OR = 2.05, 95% CI: 1.18 to 3.56, *P* = 0.011, respectively). Older age independently predicted better outcomes (OR = 1.46, 95% CI: 1.27 to 1.67, *P* < 0.001).

**Table 4:** Distribution of treatment response based on annual axial elongation and SER progression

Group	Based on AL				Based on SER				Total
	Good	Fair	Poor	*P*	Good	Fair	Poor	*P*	
DIMS	109 (44.7%)	106 (43.4%)	29 (11.9%)	< 0.001	159 (65.2%)	45 (18.4%)	40 (16.4%)	< 0.001	244
HAL	134 (71.3%)	43 (22.9%)	11 (5.9%)		146 (77.7%)	27 (14.4%)	15 (8.0%)		188
DIMSA	85 (58.2%)	46 (31.5%)	15 (10.3%)		109 (74.7%)	18 (12.3%)	19 (13.0%)		146
HALA	83 (71.6%)	30 (25.9%)	3 (2.6%)		104 (89.7%)	5 (4.3%)	7 (6.0%)		116

The data are presented as the number of eyes (percentage of total eyes in each group). The overall significance of the treatment effect on the likelihood of achieving a good response was determined using generalized estimating equation adjusted for covariates

*AL* = axial length; *DIMS* = defocus incorporated multiple segments; *DIMSA* = DIMS with 0.01% atropine; *HAL* = highly aspherical lenslets; *HALA* = HAL with 0.01% atropine; *SER* = spherical equivalent refraction

**Fig. 4:**
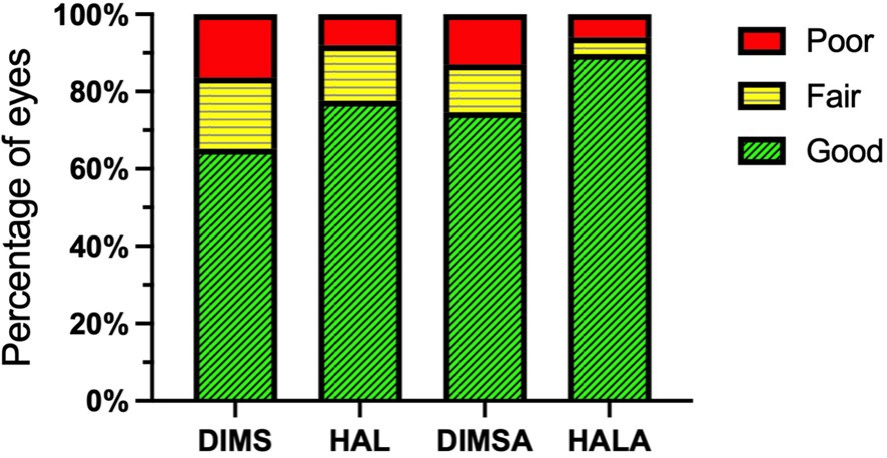
Distribution of treatment response based on spherical equivalent refraction progression. Stacked bar charts show the distribution of treatment response categories for each intervention group. Sample sizes (eyes): DIMS, n = 244; HAL, n = 188; DIMSA, n = 146; HALA, n = 116. Statistical analysis was performed using generalized estimating equation, adjusting for the same covariates used in the axial elongation response analysis. The model showed a significant overall treatment effect (Wald χ^2^ = 19.77, *P* < 0.001). The HALA group demonstrated the highest good-response rate. DIMS, defocus incorporated multiple segments; DIMSA, DIMS with 0.01% atropine; HAL, highly aspherical lenslets; HALA, HAL with 0.01% atropine

Based on SER progression, the proportion of eyes with a good response was highest in the HALA group (89.7%), followed by the HAL (77.7%), DIMSA (74.7%), and DIMS (65.2%) groups (Table 4 and Fig. 4). The GEE model confirmed a significant difference in the likelihood of a good response among the groups (Wald χ^2^ = 19.77, *P* < 0.001). Compared with DIMS, HALA was associated with the highest odds of achieving a good response (OR = 5.85, 95% CI: 2.67 to 12.82, *P* < 0.001). In contrast, neither HAL (*P* = 0.121) nor DIMSA (*P* = 0.072) showed a significant improvement over DIMS. Older age remained a significant independent predictor of a better refractive outcome (OR = 1.26, 95% CI: 1.11 to 1.44, *P* < 0.001).

In summary, HALA therapy was the most effective intervention, with HAL monotherapy also demonstrating strong effectiveness, particularly in controlling AL.

### Sensitivity analysis confirmed the robustness of primary outcomes

A sensitivity analysis was conducted using ANCOVA on the right-eye data (n = 347), adjusting for the same covariates as in the primary analysis. The results were consistent with the main findings (Supplementary Table S1 and S2). For AL, the ANCOVA revealed a significant effect of treatment (F (3, 335) = 9.52, *P* < 0.001). Axial elongation was significantly slower with HAL (MD = 0.10 mm, *P* < 0.001) and HALA (MD = 0.11 mm, *P* < 0.001) than with DIMS and with HALA than with DIMSA (MD = 0.07 mm, *P* = 0.039). The effect of treatment was also significant for SER (F (3, 332) = 4.90, *P* = 0.002). SER progression was lesser with HAL (MD: − 0.19 D, *P* = 0.003) and HALA (MD: − 0.27 D, *P* < 0.001) than with DIMS. The comparison between DIMSA and HALA showed a strong but non-significant trend favouring HALA (MD: − 0.16 D, *P* = 0.08).

Notably, the SER model included a significant group-by-age interaction (*P* = 0.041), suggesting that age may influence refractive progression outcomes. In summary, the results of this sensitivity analysis support the robustness of the primary conclusions.

## Discussion

To the best of our knowledge, this large, single-center, real-world, retrospective cohort study involved the first direct comparative evaluation of the four predominant myopia control strategies, DIMS, HAL, DIMSA, and HALA, in a clinical setting. Our findings confirmed that HAL monotherapy was associated with superior effectiveness, compared with DIMS, in an independent cohort. Although adding 0.01% atropine did not significantly improve mean axial elongation or refractive progression at the group level, its combination with HAL (HALA) significantly increased the likelihood of achieving a good response, a clinically meaningful outcome. These findings suggest that, although atropine may not enhance average effectiveness across the entire population, it may benefit a subset of children, particularly when combined with HAL. Clinicians should weigh this potential for individual response against the additional burden and side effects when considering combination therapy.

Our work addresses a specific gap in the current literature [9, 15]. Although previous studies have evaluated various combinations of optical and pharmacological therapies, no study has directly compared the DIMS and HAL optical platforms alongside their respective atropine combinations. The finding that HALA was more effective than DIMSA underscores that the choice of optical design is fundamental and that the superior baseline performance of HAL provides a more effective foundation for augmentation with low-concentration atropine.

The established superiority of HAL over DIMS as a monotherapy corroborates findings from other Chinese cohorts [6] but is in contrast with reports of equivalent performance in European populations [4]. This interpopulation discrepancy may be attributable to differences in myopia progression dynamics, genetic background, or environmental factors, underscoring the need for population-specific evaluations. The distinct optical principles of these lenses likely contribute to the differential outcomes. The highly aspherical and consistent lenslet profile of HAL may deliver a more potent and uniform defocus signal for slowing axial elongation, which could be particularly advantageous in Chinese children who often exhibit rapid progression [22].

However, this interpretation must be tempered by the recognition of important baseline imbalances. The DIMS group had significantly longer follow-up (median, 1.47 vs. 1.04–1.10 years) and more myopic baseline refraction (median, − 2.46 D vs. ≥ − 1.96 D) than the other groups did. As the effectiveness of myopia control interventions may attenuate over time, the combination of higher baseline myopia and longer follow-up duration in the DIMS group introduces uncertainty into the interpretation of its outcomes. Thus, the poorer outcomes observed in the DIMS group may partially reflect this higher-risk profile rather than an inherent inferiority of the DIMS optical design itself. Although we employed LMM to statistically adjust for these factors, such adjustments may not fully capture non-linear, time-varying effects or confounding by indication. Therefore, our findings should be interpreted as real-world associations rather than as definitive causal evidence of inherent superiority. Randomized controlled trials with balanced follow-up and baseline characteristics are needed to definitively establish comparative efficacy.

Beyond these statistical considerations, the optical mechanisms underlying DIMS and HAL lenses warrant further discussion. DIMS and HAL lenses are believed to slow myopia progression by inducing contrast modulation in specific parafoveal retinal regions, thereby creating a defocus signal that inhibits axial elongation [3, 22]. The magnitude and spatial distribution of this contrast modulation depend not only on the intrinsic optical design but also on lens power and the quality of refractive correction achieved [23]. As myopia progresses, the prescribed lenses may become suboptimal, potentially attenuating the treatment effect. In this context, repeated assessment of BCVA with the prescribed spectacles would have provided insight into the remaining treatment capacity of these lenses over time. Declining BCVA could indicate that the initial prescription no longer provides optimal correction, potentially attenuating the contrast modulation signal underlying myopia control. Unfortunately, systematic BCVA data at follow-up were not available in this retrospective cohort. Future prospective studies should include such measurements.

A pivotal finding was that adding 0.01% atropine to either DIMS or HAL did not significantly improve the mean annual AL or SER outcomes. However, this group-level result does not fully capture the clinical impact of combination therapy. In the treatment-response analysis, HALA significantly increased the odds of achieving a good response for AL (OR = 4.34) and SER (OR = 5.85) compared with DIMS, whereas this effect was not observed with DIMSA. Notably, although HALA did not significantly outperform HAL monotherapy in terms of the mean AL or SER change (*P* = 1.000 and *P* = 0.453, respectively), it demonstrated a distinct advantage in shifting children towards the good-response category. This suggests that, although the average additional benefit of atropine may be modest, it can shift a subset of children towards a more favourable clinical trajectory when combined with HAL, highlighting the importance of distinguishing between mean effects and individual response patterns when evaluating combination therapies.

Our observation that adding 0.01% atropine did not significantly improve the mean AL or SER outcomes appears to contradict previous studies that reported enhanced effectiveness for such combinations, including the superior effect of HALA over HAL [13, 14] and that of DIMSA over DIMS [9, 10]. This difference merits further exploration. The discrepancy between these findings may be explained by several factors. A central explanation lies in the concentration-dependent effectiveness of atropine. The additional effect of the 0.01% concentration may be inherently limited when added to highly effective optical interventions. This interpretation aligns with evidence from the Low-Concentration Atropine for Myopia Progression (LAMP) study, which indicated that more rapid progressors often require higher concentrations (e.g. 0.05%) for optimal effects [24]. Furthermore, a ceiling effect is possible: the potent defocus signals from HAL or DIMS may approach the maximum achievable slowing of axial elongation in some individuals, leaving minimal room for improvement with low-concentration atropine [1, 11]. In addition to concentration, differences in cohort characteristics (e.g. baseline progression rates), age distributions, and methods, such as compliance monitoring, could also contribute to the interstudy variability.

The most definitive finding of this study is the identification of HALA as the most effective regimen among the four strategies. The significant superiority of HALA over DIMSA indicates that the synergistic potential between optical interventions and low-concentration atropine is not uniform and is critically influenced by the underlying optical design. Although the primary analysis did not reveal a significant incremental benefit of HALA over HAL monotherapy, potentially due to a ceiling effect or the short-term study duration, the treatment response analysis provided crucial evidence of the clinical relevance of HALA. Compared with DIMS, it was the only regimen that significantly increased the odds of achieving a good response in terms of SER, an achievement that HAL monotherapy alone did not demonstrate. These findings suggest that the additional effect of 0.01% atropine, although modest in mean differences, may be clinically meaningful when combined with the HAL platform, by shifting more children into a category of good treatment response. However, it is important to note that these response patterns represent average effects across the entire cohort and may not capture heterogeneity in treatment response among children with different baseline progression risks. Children with rapid progression, for example, might show a different relative benefit from combination therapy, compared with slower progressors. Future studies with subgroup analyses stratified by baseline risk factors (e.g. age, baseline SER, parental myopia) are needed to determine whether the observed treatment hierarchy holds across all children or varies by risk profile.

The observed differences in axial elongation, ranging from 0.12 mm/year (HALA) to 0.23 mm/year (DIMS), represent clinically meaningful effect sizes. Compared with DIMS, HALA slowed axial elongation by an additional 0.11 mm/year, which, over 5 years, translates to approximately 0.55 mm less elongation, equivalent to roughly − 1.00 D of reduced myopia progression [25]. Even modest annual reductions can substantially lower the lifetime risk of high myopia and its associated complications. These findings support a risk-stratified approach: children with low progression risk (e.g. older age, lower baseline myopia) may do well with HAL monotherapy, whereas those with high risk (e.g. young age, rapid progression, strong family history) may benefit more from HALA as a first-line option to maximize the likelihood of achieving a good response (≤ 0.20 mm/year). DIMS may be reserved for cases in which HAL is unavailable or not tolerated. The decision to add atropine must be individualised, with potential benefits weighed against cost, side effects, and adherence.

Furthermore, our analysis of treatment predictors yielded consistent and clinically relevant insights. We found that older age was a significant independent predictor of a better treatment response for AL and SER (AL: OR = 1.46; SER: OR = 1.26). This finding aligns with the natural history of myopia progression, which typically decreases with increasing age [26, 27]. Therefore, the superior outcomes observed in older children within our cohort likely reflect this inherent biological trend, underscoring that age is a critical factor in setting expectations for myopia control therapy. Given the absence of a significant incremental benefit from adding atropine in our primary analysis, the decision to supplement HAL spectacles with 0.01% atropine in clinical practice should be carefully weighed against the additional cost, compliance, and potential side effects [28]**,** (e.g**.** photophobia and blurred near vision), as the average benefit for the general population may be limited. However, for younger children at high risk of rapid progression**,** HALA should be considered, as this subgroup is likely to achieve the greatest benefit.

This study has some limitations. First, as a retrospective, non-randomized study, treatment allocation reflects real-world selection bias, with significant baseline differences across the groups. Although we adjusted for measured covariates, residual confounding from unmeasured factors (e.g. perceived progression risk, parental preference, socioeconomic status) cannot be ruled out; thus, the findings should be interpreted as associations rather than causal effects.

Additional limitations include the following: (1) Data on environmental and genetic confounders (e.g. outdoor time, near work, parental myopia) were lacking. (2) Adherence was assessed based on parental report rather than objective monitoring, potentially diluting the effects of combination therapy. (3) Non-cycloplegic refraction was performed in a substantial proportion of eyes, with marked differences across the groups (Supplementary Table S3). To minimize potential bias in calculating progression, we applied a consistent measurement method within each individual: for eyes with cycloplegic refraction at the baseline and follow-up visits, cycloplegic measurements were used at both time points; for all other eyes (i.e. those with cycloplegic refraction at only one visit or at neither visit), non-cycloplegic refraction, performed with the fogging technique, was used at the baseline and follow-up visits to ensure internal consistency. This approach avoids introducing measurement error from combining different methods when calculating the annual SER change. Nevertheless, non-cycloplegic refraction may still introduce measurement error in the absolute SER values because of residual accommodation; however, the impact on the annual SER change is less certain. If residual accommodation remains consistent across visits, the bias may largely cancel out when calculating progression. Importantly, the primary outcome, AL, is objective and unaffected by accommodation, and the consistent ranking of treatment effects across AL and SER supports the robustness of our conclusions. (4) The use of fixed response thresholds (e.g. ≤ 0.20 mm/year for good response) does not account for age-dependent physiological eye growth and may therefore misclassify response in younger versus older children, although this bias is likely to be non-differential across groups. (5) The analyses did not account for heterogeneity in treatment response between rapid and slow progressors, as they represent average effects that may mask important subgroup differences.

Most importantly, the 1 year follow-up is insufficient to assess long-term effectiveness, potential attenuation, or rebound effects [29]. The observed treatment hierarchy may not persist with longer follow-up, and our findings should therefore be regarded as preliminary. We are continuing follow-up of this cohort and plan to report 2- and 3 year outcomes in future. Despite these limitations, our study provides valuable real-world evidence for the comparison of the four key myopia control strategies.

## Conclusions

This comprehensive real-world comparison of the four interventions confirmed that HAL spectacles were more effective as monotherapy, compared with DIMS, and showed that HALA combination therapy was the most potent strategy for myopia control in children. For clinicians, these findings suggest a potential clinical pathway: initiating therapy with HAL spectacles for strong optical control, with the option to escalate to HALA combination therapy for children requiring maximal intervention, particularly those with rapid progression or high risk of developing high myopia.

However, these findings must be interpreted with caution, given the significant baseline imbalances across the groups. The DIMS group had a longer follow-up and a more myopic baseline refraction than the other groups did, both of which may have contributed to its apparently poorer outcomes. Long-term follow-up may capture attenuation of treatment effectiveness over time, potentially underestimating the first-year effect in the DIMS group, and children with higher baseline myopia are known to progress more rapidly and may respond less favourably to treatment.

Furthermore, our treatment-response categorisation used fixed annual AL and SER thresholds that do not account for the age-dependent nature of physiological eye growth; therefore, the absolute proportions of good responders should be interpreted with caution, and age-stratified criteria may be needed for truly personalized assessment. Additionally, these findings represent average effects across the entire cohort and may not apply uniformly to all children. The optimal treatment strategy may differ between rapid and slow progressors, highlighting the need for future subgroup analyses stratified by baseline progression risk to enable truly personalized treatment recommendations.

Given the non-randomized design and potential for residual confounding, these findings should be interpreted as real-world associations rather than definitive causal effects. Future prospective, randomized trials employing cycloplegic refraction and longer follow-up are needed to confirm our findings. A critical next step is to identify predictive biomarkers or phenotypic characteristics that can distinguish optimal responders to monotherapy from those who may benefit more from combination therapy, paving the way for a truly personalized approach to myopia management.

## Supplementary Information

Supplementary material 1.

## Data Availability

The datasets used and/or analysed during the current study are available from the corresponding author on reasonable request.

## Abbreviations

AL: Axial length
ANCOV: Analysis of covariance
BCVA: Best-corrected visual acuity
CI: Confidence interval
D: Diopter
DIMS: Defocus incorporated multiple segments
DIMSA: DIMS with 0.01% atropine
GEE: Generalized estimating equation
HAL: Highly aspherical lenslets
HALA: HAL with 0.01% atropine
IQR: Interquartile range
LMM: Linear mixed model
MD: Mean difference
OR: Odds ratio
SD: Standard deviation
SE: Standard error
SER: Spherical equivalent refraction
