# Supplementary material for: Comparison of effectiveness of four myopia control interventions in Chinese children: a real-world retrospective study
Source: Eye Vis (Lond). 2026 May 12;13:18. doi: 10.1186/s40662-026-00487-z (PMC13162417; doi:10.1186/s40662-026-00487-z)
Supplement: Supplementary file 1 — Supplementary material 1. [file 40662_2026_487_MOESM1_ESM.docx]

**Supplementary materials**

**Supplementary Table S1. Sensitivity analysis (ANCOVA)**

| **Outcome** | **Group** | **Unadjusted**  **(mean ± SD)** | **Adjusted marginal mean (mean ± SE)** | ***P*** |
| --- | --- | --- | --- | --- |
| Annual AL change (mm) | DIMS | 0.24 ± 0.14 | 0.24 ± 0.01 | < 0.001 |
|  | HAL | 0.12 ± 0.17 | 0.14 ± 0.02 |  |
|  | DIMSA | 0.20 ± 0.16 | 0.19 ± 0.02 |  |
|  | HALA | 0.13 ± 0.15 | 0.12 ± 0.02 |  |
| Annual SER change (D) | DIMS | −0.44 ± 0.33 | −0.43 ± 0.04 | 0.002 |
|  | HAL | −0.22 ± 0.38 | −0.24 ± 0.04 |  |
|  | DIMSA | −0.31 ± 0.34 | −0.32 ± 0.04 |  |
|  | HALA | −0.14 ± 0.37 | −0.16 ± 0.05 |  |

AL = axial length; DIMS = defocus incorporated multiple segments; DIMSA = DIMS with 0.01% atropine; HAL = highly aspherical lenslets; HALA = HAL with 0.01% atropine; SD = standard deviation; SE = standard error; SER = spherical equivalent refraction

This sensitivity analysis was performed using analysis of covariance (ANCOVA) on the right-eye data only (n = 347) to confirm the robustness of the primary linear mixed model findings. The model was adjusted for age, sex, follow-up interval, and baseline ocular parameters (AL, SER, cylinder power, corneal astigmatism, and corneal curvature).

**Supplementary Table S2. Sensitivity analysis (ANCOVA) for treatment group comparisons**

| **Comparison** | **Annual AL change** | | **Annual SER change** | |
| --- | --- | --- | --- | --- |
|  | **MD (95% CI)** | ***P*** | **MD (95% CI)** | ***P*** |
| DIMS vs. HAL | 0.10 (0.04 to 0.16) | < 0.001 | −0.19 (−0.33 to −0.05) | 0.003 |
| DIMS vs. HALA | 0.11 (0.05 to 0.18) | < 0.001 | −0.27 (−0.43 to −0.11) | < 0.001 |
| DIMSA vs. HALA | 0.07 (0.002 to 0.14) | 0.039 | −0.16 (−0.33 to 0.01) | 0.08 |

AL = axial length; CI = confidence interval; DIMS = defocus incorporated multiple segments; DIMSA = DIMS with 0.01% atropine; HAL = highly aspherical lenslets; HALA = HAL with 0.01% atropine; MD = mean difference; SER = spherical equivalent refraction

Post hoc pairwise comparisons from the ANCOVA model (right-eye data only) with Bonferroni correction. Only comparisons that yielded significant results are shown; non-significant comparisons are omitted.

**Supplementary Table S3. Proportion of eyes undergoing cycloplegic refraction at the baseline and follow-up visits**

| **Group** | **Baseline** | **Follow-up** |
| --- | --- | --- |
| DIMS | 99.2% (242/244) | 59.8% (146/244) |
| HAL | 92.6% (174/188) | 42.6% (80/188) |
| DIMSA | 68.5% (100/146) | 35.6% (52/146) |
| HALA | 87.9% (102/116) | 48.2% (56/116) |

DIMS = defocus incorporated multiple segments; DIMSA = DIMS with 0.01% atropine; HAL = highly aspherical lenslets; HALA = HAL with 0.01% atropine

Data are presented as percentages (number of eyes undergoing cycloplegic refraction/total number of eyes in the group).
